# Chemistry-Induced Effects on Cell Behavior upon Plasma Treatment of pNIPAAM

**DOI:** 10.3390/polym14061081

**Published:** 2022-03-08

**Authors:** Veronica Satulu, Valentina Dinca, Mihaela Bacalum, Cosmin Mustaciosu, Bogdana Mitu, Gheorghe Dinescu

**Affiliations:** 1 National Institute for Lasers, Plasma and Radiation Physics, Atomistilor 409 Street, Magurele, P.O. Box MG-36, 077125 Bucharest, Romania; veronica.satulu@inflpr.ro (V.S.); valentina.dinca@inflpr.ro (V.D.); gheorghe.dinescu@inflpr.ro (G.D.); 2 Horia Hulubei National Institute of Physics and Nuclear Engineering—IFIN HH, 30 Reactorului Street, Magurele, P.O. Box MG-36, 077125 Bucharest, Romania; bmihaela@nipne.ro (M.B.); cosmin@nipne.ro (C.M.)

**Keywords:** pNIPAAM, plasma treatment, surface chemistry, carboxylic and amide bonds, cytocompatibility, cells attachment/detachment

## Abstract

In the field of bioengineering, depending on the required application, the attachment of various biological entities to the biomaterial is either favored or needs to be prevented. Therefore, different surfaces modification strategies were developed in combination with the characteristics of the materials. The present contribution reports on the use of the specific surface property of a thermoresponsive polymer poly(N-isopropylacrylamide) pNIPAAM obtained by spin coating in combination with plasma treatment for tuning cell behavior on treated polymeric surfaces. Topographical information for the plasma-treated pNIPAAM coatings obtained by Atomic Force Microscopy (AFM) measurements evidenced a more compact surface for Ar treatment due to combined etching and redeposition, while for oxygen, a clear increase of pores diameter is noticed. The chemical surface composition as determined by X-ray Photoelectron Spectroscopy showed the specific modifications induced by plasma treatment, namely strong oxidation for oxygen plasma treatment illustrated by eight times increase of O-C=O contribution and respectively an increase of C-N/O=C-N bonds in the case of ammonia plasma treatment. Structural information provided by FTIR spectroscopy reveals a significant increase of the carboxylic group upon argon and mostly oxygen plasma treatment and the increase in width and intensity of the amide-related groups for the ammonia plasma treatment. The biological investigations evidenced that L929 fibroblast cells viability is increased by 25% upon plasma treatment, while the cell attachment is up to 2.8 times higher for the oxygen plasma-treated surface compared to the initial spin-coated pNIPAAM. Moreover, the cell detachment process proved to be up to 2–3 times faster for the oxygen and argon plasma-treated surfaces and up to 1.5 times faster for the ammonia-treated surface. These results show the versatility of plasma treatment for inducing beneficial chemical modifications of pNIPAAM surfaces that allows the tuning of cellular response for improving the attachment-detachment process in view of tissue engineering.

## 1. Introduction

Recently, there is an increased interest in the development of materials whose surface properties can be dynamically tuned by external stimuli to ensure additional functionality. These stimuli can be classified as chemical (e.g., pH, ionic factors and chemical agents) or physical, such as, temperature, light, and/or magnetic field [1]. Chemical stimuli induce interactions at the molecular level, either polymer–polymer or polymer–solvents, while physical stimuli affect the molecular interactions at critical onset points [2]. Such materials are often called “stimuli-responsive polymers” or “smart polymers (SP)” or “intelligent polymers” [3]. The control of the surface properties allowed by their responsive nature are of great importance for bioengineering and biotechnology applications [1].

For biomedical applications, the systems that are sensitive to temperature or pH are the most important due to the polymer–polymer and polymer–solvent interactions which indicate a sudden readjustment for small ranges of pH or temperature modifications, often related to the collapse or extension of the polymeric chains [4]. Moreover, the temperature is the most widely used stimulus for “smart polymers” due to the easiness of the control and for the facile applications in vitro and in vivo [5]. The polymers that possessed the ability to respond to a change in the temperature can be classified into two main types: the systems that become insoluble upon heating are thermoresponsive polymers (TRPs), possessing lower critical solution temperature (LCST), and the systems that become soluble upon heating are TRPs, possessing upper critical solution temperature (UCST) [6]. 

From the first report of Heskins and Guillet [7] on the poly(N-isopropyl acrylamide) (pNIPAAM) behavior with temperature, which opened up the field of the so-called smart materials, several compounds have been evaluated to understand the paths for modifications of critical solution temperature, both among polymers presenting LCST, like poly(oligo(ethylene glycol)ethyl ether methacrylate (POEGMA), poly(N,N-dimethylaminoethylmethacrylate) (PDMAEMA) or poly(4-vinylpyridine) (P4VP) [8], but also evidencing UCST, as poly(acrylic acid) (PAA), polyacrylamide (PAAm), and poly(acrylamide-co-butyl methacrylate) are [9]. Nevertheless, pNIPAAM remained of particular interest in the field of bioengineering due to the phase change that it undergoes in a physiologically relevant temperature range, almost independent of its concentration, which favors the cell or protein release [10]. The lower critical solution temperature (LCST) for pNIPAAM is 32 °C in water, having a collapsed hydrophobic state above this temperature and an extended hydrophilic state below LCST [11]. 

The thickness of the pNIPAAM layers (<30 nm) plays an important role in temperature-induced hydrophilic/hydrophobic state and cell attachment above LCST and detachment below LCST. Numerous studies indicate that the surface of pNIPAAM layers with thicknesses higher than several tens of nm inhibits cell adhesion. In addition, highly porous surfaces inhibit the formation of the cells’ monolayer, interfering in the attachment-detachment process [6]. Among the most successful methods for optimizing the thermoresponsivity of pNIPAAM is the usage of polymeric brushes covalently linked to the surface, via various grafted peroxides on the substrate [12], and pNIPAAM functionalization with carboxylic groups [13], while poly(NIPAM-co-2-carboxyisopropylacrylamide) copolymers showed its efficiency in molecules separation and cell harvesting.

An optimal pNIPAAM layer for cell seeding involves complex and non-easily fabrication methods, such as covalently graft pNIPAAM chains by electron beam irradiation [14]. Other research indicates the successful utilization of the Matrix-Assisted Pulsed Laser Evaporation technique (MAPLE) for the pNIPAAM with controlled protein adsorption and cells reversible attachment [15]. The spin-coating technique represents a simpler and more efficient method for manufacturing pNIPAAM layers, which can serve as a thermoresponsive surface for cell culture and cell sheet harvesting [16]; however, in this case, the obtaining of very thin layers might be difficult, additional processing being often necessary.

Utilization of plasma for producing pNIPAAM thin films showed significant advantages in ensuring the desired thickness as well as additional functional groups on the surface that may enhance the attachment–detachment kinetics [17]. The advantages of plasma come from its constituents, active species, radicals, electrons and ions, together with the radiation induced within the system, which free the polymerization process from any solvents or chemical initiators [18] or additional purification steps. Moreover, these constituents individually contribute to the final properties of the deposits via specific interactions [19]. At the same time, the plasma ability to activate substrates for further grafting [20] or plasma graft polymerization [21] were previously reported for the synthesis of very thin layers of pNIPAAM layers. Plasma was also successfully used for surface functionalization with various chemical groups, inducing hydrophobic [22] or hydrophilic [23] behavior, evidencing the versatility of processes as a tool for tissue engineering [24]. As such, the combination of pNIPAAM spin coatings [16] with a mild plasma treatment to superficially modify its surface represent a good strategy for optimization of surface cell engineering. Until now, scarce research has been reported on the behavior of plasma-treated pNIPAAM surfaces [25], without evaluating various plasma environments or soft plasma treatment. 

In this study, we report on the tuning of the pNIPAAM spin-coated thin films properties by short plasma exposure in different environments, namely in argon, oxygen, and ammonia, showing that such approach properly modulates the material surface for the attachment/detachment of cells, regardless the film thickness. We evidence that cell viability can be significantly improved upon plasma treatment, while the cell attachment/detachment is particularly favored by the pNIPAAM surface oxidation.

## 2. Materials and Methods

### 2.1. Samples Preparation

**Figure 1:**
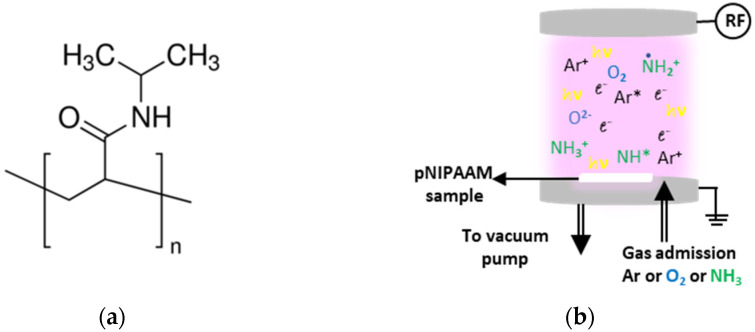
(**a**) Chemical structure of pNIPAAM. (**b**) Experimental set-up used for plasma treatment of pNIPAAM.

Round glass coverslips (1.3 cm^2^) and 1 cm^2^-square double-polished Si (100), transparent in IR (provided by Neyco), were used as substrates for the biological investigations and morpho-chemical characterization, respectively. They were carefully cleaned in an ultrasonic bath in acetone, ethyl alcohol, and deionized water and blow-dried with N_2_ gas before use. pNIPAAM solution was prepared by dissolving the polymer, having the chemical structure presented in Figure 1a, into chloroform, at 1% weight concentration, under vigorous stirring. All reagents were purchased from Sigma-Aldrich and used as provided unless otherwise specified. The coatings were prepared by dropping 200 μL of this solution onto coverslips, respectively Si substrates, and spin-coated in a single step for 60 s at 2000 rpm using a Laurell Tech spin coater (Laurell Technologies, North Wales, PA, USA), conducting to a uniform pNIPAAm coating of 330 nm thickness.

The plasma treatment experiments were conducted in a glass vacuum chamber, which is pumped down by a mechanical pump to a base pressure of 2.5 × 10^−2^ mbar (Figure 1b). The chamber is provided with two parallel aluminum electrodes, separated by 6 cm distance, where the upper one is RF active and the lower is grounded, serving as substrate holder as well. Various gases, namely argon, ammonia and oxygen, were used for treatment in order to induce different effects on the pNIPAAM surface. They were introduced in the reactor at constant flow of 30 sccm by means of Bronkhorst mass flow controllers, so the established working pressure was around 0.7 mbar. The plasma treatment of pNIPAAM was carried out at 20 W RF power (13.56 MHz RF generator, model AX-600 III-A-NV1, ADTEC Plasma technology Co., Fukuyama, Hiroshima, Japan) capacitively coupled via an automatic matching box model AMV-1000-EN ADTEC Plasma technology Co., Fukuyama, Hiroshima, Japan) for a set time of 30 s. 

### 2.2. Material Investigations

The surface topography of thin films was investigated by Atomic Force Microscopy (AFM), using a Park Systems XE-100 apparatus (Suwon, Korea), working in non-contact mode, for areas ranging from 10 µm × 10 µm down to 2 µm × 2 µm.

The determination of apparent pores diameter was conducted by using ImageJ software (1.51j8 version, National Institutes of Health (NIH Image), Washington, DC, USA, http://rsb.info.nih.gov/ij/, accessed on 23 January 2022), designed for scientific multidimensional images processing. The average apparent pores diameter and the standard deviation were determined from 50 measurements on each sample, allowing relevant statistics on the data.

The contact angle investigations were carried out by using a Kruss drop-shape analyzer model DSA 100S (Up-grade) provisioned with a software-controlled dosing system and a temperature-controlled chamber TC 40 with a Peltier element accompanied by a PT 100 temperature sensor for ensuring measurements both below and above LCST. In particular, the experiments were conducted by dropping 2 μL distilled water on the initial and plasma-treated pNIPAAAM surfaces, kept at a constant temperature of 22 °C and respectively 37 °C. The reported results are the average of 5 drops on each substrate temperature, as determined by circle fitting 1 s after reaching the surface.

The chemical structure of the initial and argon, ammonia and oxygen plasma-treated pNIPAAM coatings was determined by Fourier Transform Infrared spectroscopy (FTIR) measurements using a JASCO 6300 spectrometer (Easton, MD, USA), equipped with a transmission module. The FTIR spectra were recorded in the range of 400–4000 cm^−1^, with a resolution of 4 cm^−1^ and an average number of 512 scans. 

The chemical composition of the porous pNIPAAM thin film surfaces was investigated by using the X-ray Photoelectron Spectroscopy (XPS) method. XPS analyses were performed on a K-Alpha Thermo Scientific (ESCALAB™ XI+, East Grinstead, UK) spectrometer equipped with a 180° double-focusing hemispherical analyzer. The calibration of the peak positions was made with respect to the standard C1s peak (284.8 eV). Survey spectra were recorded at a pass energy of 50 eV to determine the surface elemental composition. High-resolution spectra for C1s, O1s and N1s binding energy regions were measured at pass energy of 20 eV in order to evaluate the elemental bonding states of the as-functionalized materials. The spectra acquisition and data processing were performed by using the advanced Avantage data software ( Thermo Avantage v5 9921, East Grinstead, UK). 

### 2.3. In Vitro Studies

The samples sterilization was performed in two steps, as previously reported in [26], by placing the glass substrates coated with pNIPAAM on 70% alcohol wet paper under a UV lamp (Philips G30T8, Eindhoven, The Netherlands) for one hour and, in the second step, by soaking and washing the samples in sterile serum-free Hank’s balanced salt solution (SF-HBSS, Thermo Fisher catalog no. 14170-122).

Prior to seeding on the samples, the cells were detached with trypsin (Merck catalog no. T4049), resuspended in Minimum Essential Medium (MEM, Merck catlog no. D5671) and afterwards brought to a concentration of 2 × 10^6^ cells/mL. A volume of 15 μL of this suspension was uniformly placed onto the sample’s surface, and then 1.5 mL of medium was added to each well. The plate was incubated at 37 °C, 5% CO_2_ in a humid atmosphere for 48 h (SafeGrow Pro incubator–Euroclone, Italy). After that, the samples were moved to other wells, and 400 μL of viability solution (CellTiter 96^®^ AQueous Non-Radioactive Cell Proliferation Assay (MTS)–Promega) was added (20 µL MTS at 100 µL MEM solution). The plates were incubated for 3 h at 37 °C, 5% CO_2_ in a humid atmosphere, movements of the plates leading to homogeneous MTS solution and reduction of the active element. After the incubation, the MTS solution was transferred into a 96 well plate and absorption at 490 nm was read by a TECAN Sunrise Basic plate-reader (Tecan Austria GmbH, 5082 Grödig, Austria).

The cells’ behavior was evaluated by means of fluorescence microscopy. As such, the cells were colored with acridine orange solution (Merck catalog no. A6014) for 15 min. All the stained samples were washed twice with sterile SF-HBSS medium and then analyzed with a fluorescence microscope (Olympus IX71-Olympus Corporation, Tokyo, Japan) for cells morphological characteristics, respectively with an Olympus microscope (CKX31-CKX31SF, Olympus Corporation, Tokyo, Japan) for analyzing the detachment of the cells. Fluorescence images were taken using a CCD camera (ANDOR iXon DU897 E-CSO-UVB), while the cells detachment images were taken with a Watec camera (WAT-902H, Tokyo, Japan). Thehe image analysis and counting were performed by using the grayscale representations of each field and ImageJ software.

## 3. Results

### 3.1. Material Characterization

#### 3.1.1. Topographical Characterization of the Plasma Treated pNIPAAM Coatings

**Figure 2:**
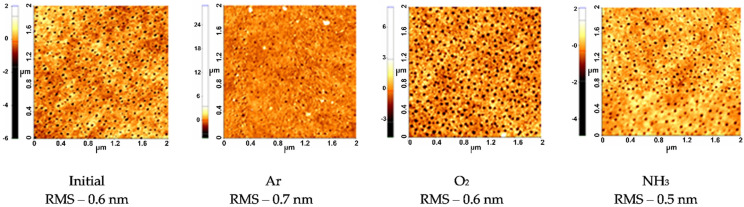
AFM images of the PNIPAAM coatings initial and upon plasma treatment of 30 s performed in various gases, at 20 W applied RF power and 0.5 mbar pressure.

In order to evidence the surface evolution with respect to the topographical aspects for the untreated and various gases plasma-treated pNIPAAM thin films, AFM measurements performed on 2 × 2 μm areas are presented in Figure 2. The untreated pNIPAAM coatings present a very smooth surface with a roughness RMS of only 0.6 nm. Very small circular pores with diameters smaller than 30 nm are observed on the surface, most probably formed upon fast solvent evaporation taking place during the spin coating deposition of pNIPAAM thin films. Very smooth surfaces, with roughness RMS values below 1 nm, were determined as well upon RF plasma treatment for all investigated gases, suggesting that RF plasma treatments induced insignificant modification of the material surface roughness. Nonetheless, the surface aspect changes with respect to the pores’ appearance according to the type of plasma used. 

**Figure 3:**
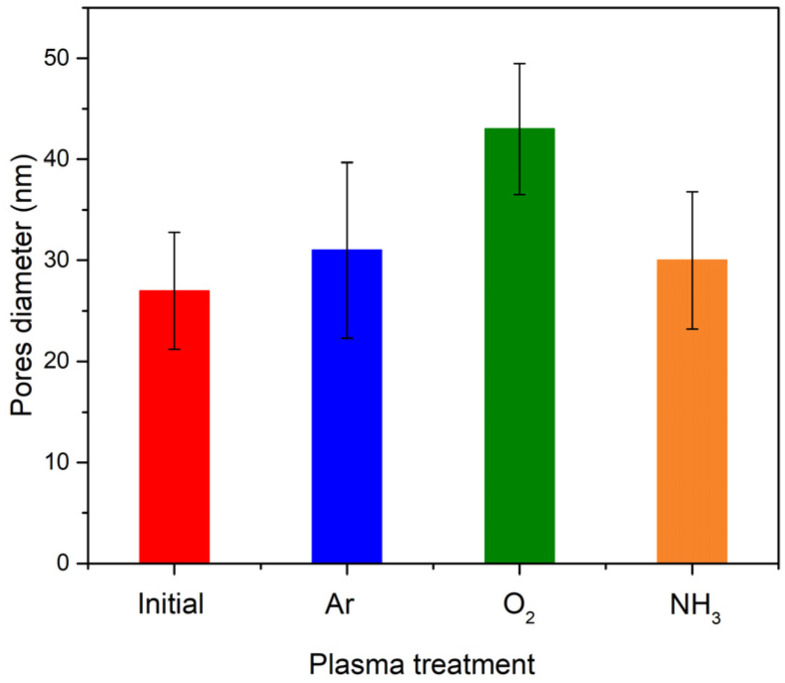
Variation of pores diameter upon plasma treatment.

As such, Figure 3 are presented the modifications of the diameter of the apparent pores as measured on the untreated and plasma-treated pNIPAAM surfaces in respect to plasma treatment, evidencing larger pores in all cases. In the case of oxygen plasma-treated pNIPAAM layers, the topographical aspects indicate an increasing of apparent pore’s size up to 42 nm, without any significant effects regarding the surface roughness. These changes are due to the well-known etching effect of the oxygen species, both atoms and ions, present in the discharge over the polymeric surface [27]; indeed, an etching rate of pNIPAAM coating of 0.74 nm/s was determined in these plasma conditions. In the case of argon plasma treatment of the pNIPAAM layers, the etching rate was lower than for the oxygen plasma case, of just 0.34 nm/s, and the surface revealed a more compact aspect. As such, we consider that in argon plasma, a combined mechanism of pNIPAAM sputtering conducted via higher mass Ar ions followed by redeposition of the freshly polymeric fragments and the surface rearrangement in a more compact manner is taking place in this case. Regarding the ammonia plasma treatment of the pNIPAAM surface, a very low rate of removal of only 0.06 nm/s was determined, and only a slight increase of the apparent pore’s diameter in the pNIPAAM materials with more aerated aspects was observed, suggesting just a surface functionalization without any significant modification of surface features. 

#### 3.1.2. Chemical Composition of the Plasma Functionalized pNIPAAM Coatings

**Figure 4:**
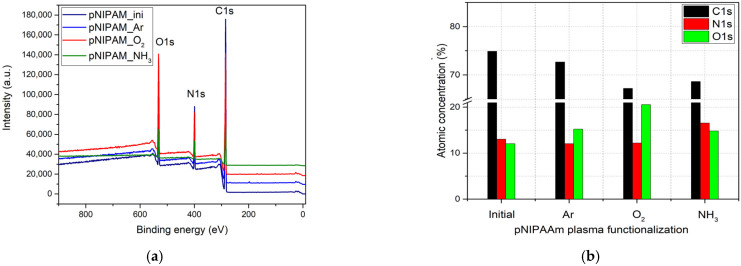
Survey spectra for initial and plasma-treated pNIPAAM coatings (**a**); variation of the atomic concentration of the main elements regarding the gas plasma treatment (**b**).

An X-ray Photoelectron Spectroscopy (XPS) wide scan (Figure 4a) revealed on the untreated and various gases plasma functionalized pNIPAAM surfaces the presence of carbon, nitrogen, and oxygen as the main elements which are specific to pNIPAAM materials. The dependence of the atomic contribution of elements as a function of various plasmas used for functionalization, as quantified from the XPS survey spectra processing, are presented in Figure 4b. 

The contribution related to C1s line decreases after plasma treatment, more pronounced in the case of oxygen plasma treatment, suggesting on one side the carbon atoms removal from the pNIPAAM surface and on the other side the mighty oxidation effect both in the plasma media and in the ambient surrounding. The O1s signal presents an opposite behavior in respect to C1s line, increasing after plasma treatment regardless of the used plasma, but with an important contribution in the case of oxygen plasma treatment. This behavior suggests that some of the carbon atoms are removed from the surface and additional bonding with oxygen appears on the pNIPAAM surface, suggesting an oxidation effect occurring both in the plasma media and in the ambient surrounding. Regarding the N1s line, the atomic concentration is slightly decreasing in the case of argon and oxygen plasma treatment, suggesting the partial removal of nitrogen-related bonds, while it presents an important contribution in the case of ammonia plasma treatment that shows successful functionalization of pNIPAAM surface after plasma exposure, with predictable surface chemical composition with respect to the working gas used in the discharge. This presents an unimportant variation in the case of argon and oxygen plasma treatment, pointing out the preferential affinity of the plasma species in respect of working gas used in the discharge. Therefore, we can conclude here that carbon and oxygen are the key elements for the plasma etching processing in argon and oxygen atmosphere, while in the case of ammonia plasma, the higher contribution of nitrogen is related to the functionalization of the pNIPAAM surface by attaching the -NH groups to free radicals resulted from molecules collisions with the electrons present in the discharge, even in the case of soft RF power using.

**Figure 5:**
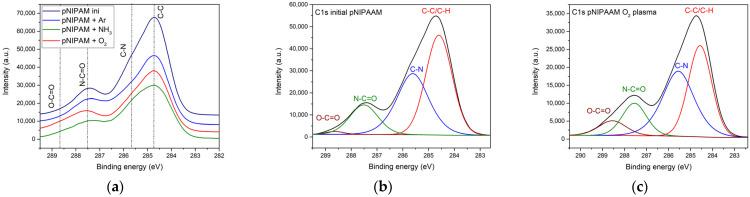
(**a**) Comparative high-resolution C1s spectra for various gases plasma treatment; (**b**) high-resolution spectrum of C1s binding energy region for the initial pNIPAAM; (**c**) high-resolution spectrum of C1s binding energy region in the case of oxygen plasma treatment.

The high-resolution C1s spectra recorded for the initial pNIPAAM coating and the RF various gases plasma-treated pNIPAAM surfaces are displayed in Figure 5a. The deconvolution of C1s spectrum of the initial pNIPAAM thin film was performed using four components, as follows: C-C/C-H at 284.6 eV, C-N at 285.7 eV, N-C=O at 287.5 eV and O-C=O bond at 288.7 eV, and is illustrated in Figure 5b. The slight variations of spectra noticed in the C1s spectra of the various gases’ plasma treatment of the pNIPAAM surface could be quantitatively evaluated upon deconvolution also in four components, corresponding to the presence of the C-C/C-H (284.6 eV), amine groups C-N (285.6 eV), amide bonds N-C=O (287.5 eV), and O-C=O (288.7 eV) chemical bonds in the plasma-processed pNIPAAM structure [17]. An example of such deconvolution is shown in Figure 5c in the case of oxygen plasma treatment.

**Figure 6:**
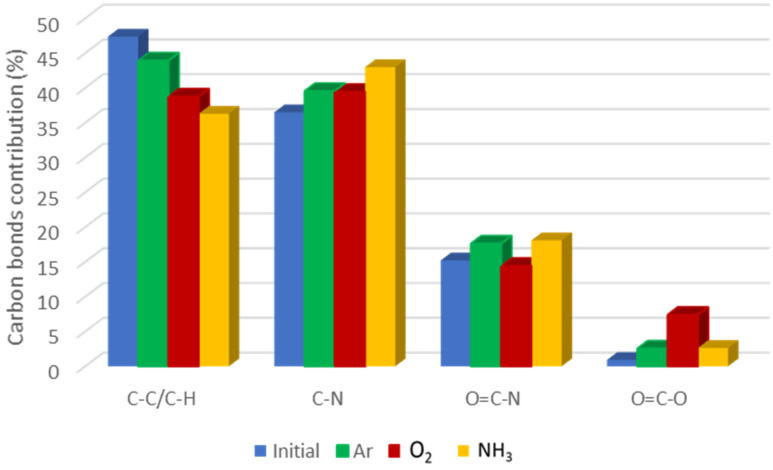
Comparative carbon bonds contribution in the pNIPAAM layers depending on plasma treatment working gas.

In Figure 6, the relative contribution of various carbon bonds to the overall C1s-related XPS signal is shown, depending on the gas utilized during plasma treatment in various gases of pNIPAAM surface. It shows that in the case of plasma treatment of pNIPAAM surface, the contribution of C-C bonds is decreasing regardless of the plasma ignition gas type and is more pronounced for the ammonia plasma treatment, suggesting the reactive character of this gas. Regarding the C-N bonds, these present an opposite behavior, increasing for all investigated gases, with an important contribution in the case of ammonia plasma treatment, suggesting that two complementary processes are taking place, physical desorption due to the etching process followed by chemical deposition due to the free surface bonds satisfaction with plasma species. Slight oxidation can be observed even for the initial spin-coated pNIPAAM, with a contribution below 1%, most probably due to slight absorption of water molecules from the ambient surrounding. The significant increase of O-C=O contribution, from 0.92% for the initial pNIPAAM coating to 7.39% for the case of oxygen plasma treatment, clearly shows that plasma treatment favors the formation of carboxylic groups on the pNIPAAM surface and therefore promote the cellular attachment in this case. The results show cross-linking of the pNIPAAM at the surface as the result of the mild plasma treatment applied, regardless of the type of gas and specific functionalization, which strongly depends on the gas, the carboxylic bonds being predominant for the oxygen while the amination/amidation is prominent in the case of ammonia plasma.

**Figure 7:**
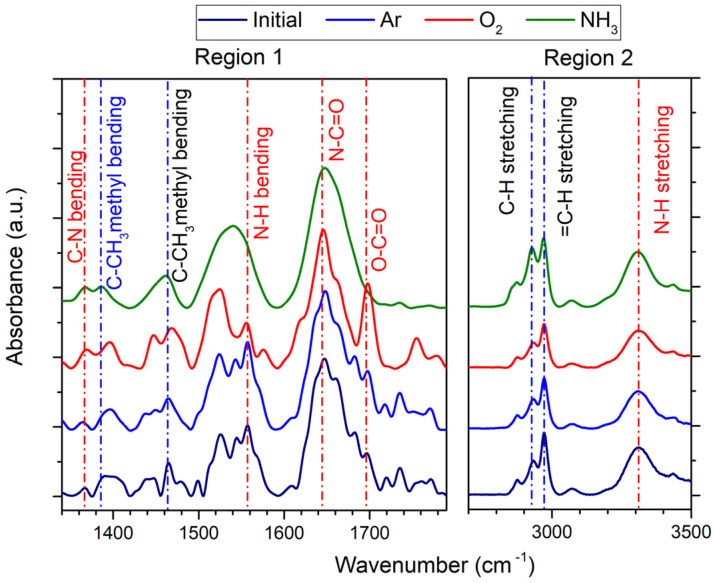
FTIR spectra of untreated and treated pNIPAAM material in argon, oxygen, and ammonia plasmas.

FTIR technique allowed complementary investigation of the chemical bonds of the pNIPAAM coatings functionalized by 30 s exposure to RF plasma ignited in various gases, namely argon, oxygen, and ammonia. The FTIR spectra recorded for the plasma-treated pNIPAAM thin films together with the spectrum of the untreated pNIPAAM coating are presented in Figure 7. Typical FTIR spectra of PNIPAAM materials reveal the presence of specific bonds, detailed in Table 1, corresponding to hydrophilic amides bonds, highlighted in red color, and hydrophobic isopropyl groups, highlighted in blue color. Notable peaks are assigned as follows: secondary amides, N-C=O stretching of amide I bond at 1646 cm^−1^, N-H bending of amide II bond at 1540 cm^−1^, C-N stretching vibration at 1367 cm^−1^ [15], isopropyl methyl -CH(CH_3_)_2_) bending vibrations at 1388 cm^−1^ and 1460 cm^−1^ [28], CH_3_ symmetric and asymmetric stretching modes at 2874 cm^−1^ and 2970 cm^−1^, and CH_2_ asymmetric stretching vibration at 2934 cm^−1^ [6,29]. 

Additionally, in the high wavenumber region, one can notice the presence of secondary amide N-H stretching around 3308 cm^−1^ and the band at 3435 cm^−1^ associated with free N-H stretching [30]. The same peaks are evidenced in the structure of the plasma-treated pNIPAAM coating with peculiar intensity depending on the gas injected in the discharge. One can notice the strong absorption band situated around 1700 cm^−1^ assigned to the carboxylic group in the case of oxygen plasma ignition in correlation to the XPS data that showed the strong increase of O-C=O bonds. This suggests an important improvement of the surface wettability.

The specificity of the plasma treatment is also evidenced in the case of ammonia plasma ignition by the strong and broad absorption band corresponding to the polar -NH group, ensuring the affinity of the surface for water. The conservation of hydrophobic groups, as evidenced in all FTIR spectra, corroborated with the preservation of hydrophilic linkages ensure the hydration/dehydration structure of the pNIPAAM coatings and thus the attachment and detachment of the cells [31]. 

### 3.2. Investigation of Wettability Behavior with the Temperature

**Figure 8:**
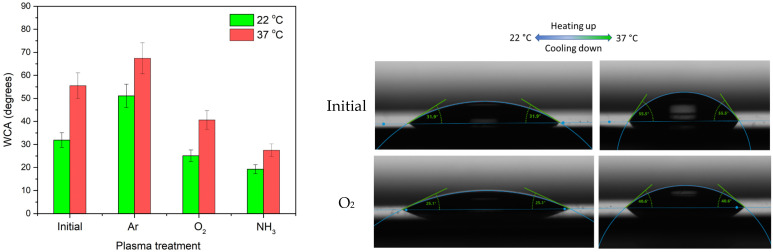
**Left**—WCA at 22 °C (below LCST) and 37 °C (above LCST) for the spin-coated pNIPAAM and plasma-treated surfaces in argon, oxygen and ammonia plasmas; **Right**—images of the droplets on the surface of initial and oxygen plasma-treated pNIPAAM, below and above LCST.

In order to assess the wettability behavior, water contact angle measurements have been performed at room temperature (22 °C), associated with the lowest detachment temperature, and respectively at 37 °C, corresponding to the cell incubation/attachment temperature. The results, presented in Figure 8, indicate for all the surfaces a lower contact angle at low temperature, and a higher contact angle above LCST, evidencing that the surface thermoresponsiveness is maintained in all the investigated conditions. While lower contact angles are obtained in the case of reactive oxygen and ammonia plasma, correlating to the surface topography and functionalization with carboxyl and amine groups, respectively, higher contact angles were measured in the case of Ar plasma treatment, which supports the AFM measurements showing a more compact surface in this case.

At the same time, the gradient of the water contact angle encountered between the hydrated state and the collapsed one is the highest for the initial pNIPAAM, correlating to a strong influence of the bulk polymer, and the lowest for the NH_3_ plasma treatment, indicated in this last case a lower ability to expand/fold. These results show the combined role of surface topography and functional groups in obtaining the desired properties, and pave the way towards development of multiple stimuli-responsive surfaces, combining the response to pH to that previously determined for temperature [32]. 

### 3.3. Biocompatibility Assessment of pNIPAAM Surfaces Treated with Various Gases Plasma

#### 3.3.1. In Vitro Biological Investigations

**Figure 9:**
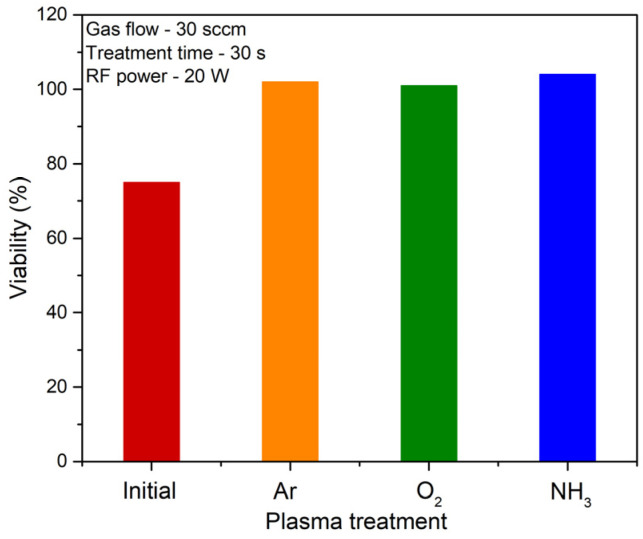
Cell viability for the L929 fibroblasts grown on the PNIPAAM initial surface as obtained by spin coating and respectively treated in argon, oxygen and ammonia plasma.

According to the MTS assays presented in Figure 9, cells proliferated at T = 37 °C onto the PNIPAAM films treated by plasma with viability increased by more than 25% as compared with the control surfaces (not treated). A similar increase in percentage was observed for all the pNIPAAM surfaces treated with argon, oxygen and ammonia, suggesting a clear improvement in cell viability due to the presence of functional groups onto film surfaces, regardless the type of treatment. As such, we relate this behavior to the increase of the carboxylic groups on the surface, observed for all the investigated cases, while the contribution of amines and N-C=O groups induced upon ammonia treatment should also be considered responsible. 

Nevertheless, the main differences were given by cell morphology and cell number adhered onto the initial and plasma-treated surfaces. For this, L929 cell diameter and occupied area were evaluated as a function of treatment type to gain an understanding of attachment efficiency.

**Figure 10:**
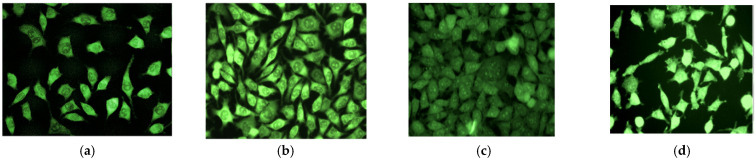
Images of the L929 fibroblast cells growth on the untreated pNIPAAM (**a**); argon (**b**), oxygen (**c**) and ammonia (**d**) plasma-treated pNIPAAM surface.

It was shown that after 48 h the cell diameters and spreading were similar for O_2_ and Ar, the cells showing a normal morphology, flat and spread, while for ammonia, a lower number of attached cells are observed attached. From the fluorescence micrographs of the fixed and stained cells presented in Figure 10, it can be concluded that although cells showed normal morphology, flat and spread for all the three gases considered for the polymer plasma treatment for 0.5 mbar, 20 W, 30 s, a difference in the number of cells adhered onto the treated PNIPAAM films was observed, suggesting the importance of the chemical groups onto the surface. Although NH_3_ was reported as it would be favorable for higher adhesion, in our case, it was showing better adhesion than control pNIPAAM samples, but a lower number of cells adhered as compared with O_2_ and Ar-treated surfaces. One of the main issues could be related to stability in a time of the amino groups, which can also provide an explanation for our results [33].

#### 3.3.2. Cell Detachment Studies

**Figure 11:**
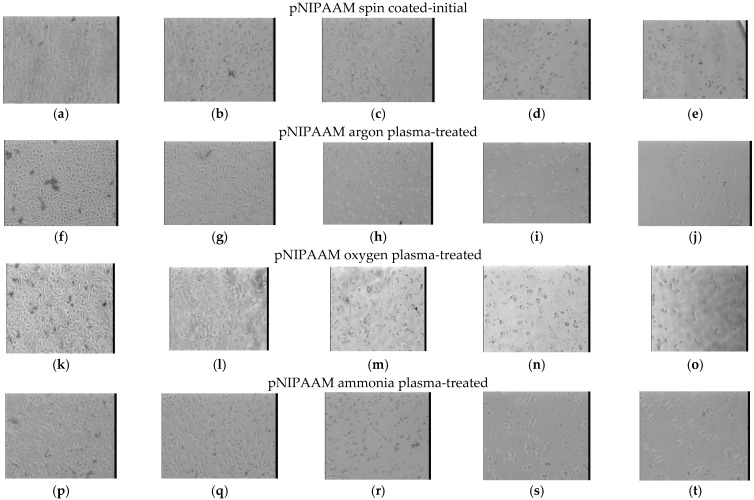
Cell detachment sequence images of PNIPAAM surfaces, initial and treated in argon, oxygen and, respectively, ammonia plasma for 30 s, initial (**a**,**f**,**k**,**p**) 0 min; and respectively upon cooling, after: (**b**,**g**,**l**,**q**) 15 min; (**c**,**h**,**m**,**r**) 25 min; (**d**,**i**,**n**,**s**) 35 min; and (**e**,**j**,**o**,**t**) 45 min.

For the detachment studies, the first set of phase images presented in Figure 11 in the left column shows the morphology of the cells on the pNIPAAM thin films under normal culture temperature (37 °C), corresponding to conditions implying temperatures above the LCST of the pNIPAAM polymer. The cells were attached to the surface and were proliferating. It is clear that no cells detach from any of the plasma-treated pNIPAAM or the control samples (both pNIPAAM and glass) within the experimental time frame. These results are consistent with previous reports that in order to stimulate detachment, the solution used must be below the LCST of the polymer [34].

As such, the next columns in Figure 11 are associated with the behaviour of the cells on the pNIPAAM surfaces, initial and plasma-treated, upon decrease of temperature in time, for an interval of 45 min, down to room temperature (22 °C), and such corresponding to below LCST. When the pNIPAAM growth support was cooled down, the morphology of the cells was less spread and rounder, as shown in the next panel of Figure 11b,g,l,q. The detachment induced by the temperature decrease and hydration of pNIPAAM chains continues as shown in Figure 11 to the fourth (d,i,n,s) and fifth set (e,j,o,t) in which the cells no longer have a spread, flattened morphology and they detached in a high percentage (more than 75% as compared with starting ones). Most of the cells rounded and started to float off the substrate within 30–45 min, and the few remaining cells were characterized by highly circular shapes. 

**Figure 12:**
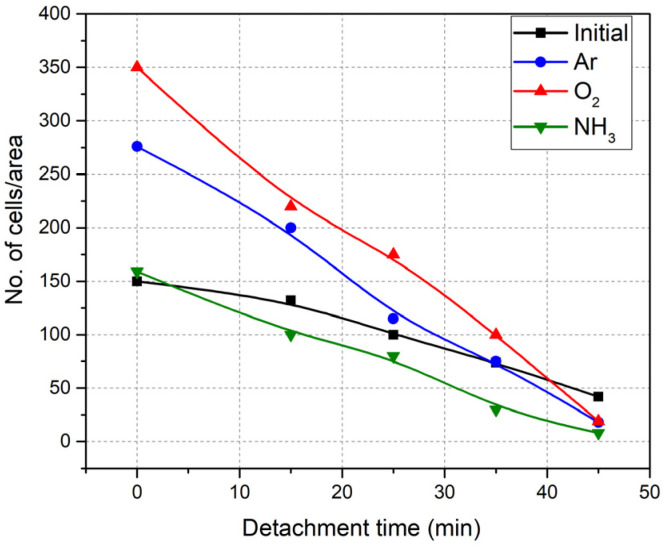
Number of cells present on the surface as a function of cooling time, from 37 °C to 22 °C, for the initial and plasma-treated pNIPAAM surfaces.

The results regarding the number of cells remaining on the surface in this time interval, are presented in Figure 12. As a general remark, it is noticed that the cell detachment was more pronounced for all the plasma-treated surfaces with respect to the non-treated spin-coated pNIPAAM surface. Of the surfaces tested, cells attachment onto pNIPAAM surfaces treated in argon and oxygen plasmas was highest, being 2.3 and 1.8 times higher for the oxygen and argon plasma-treated surfaces, respectively, when compared to non-treated pNIPAAM one; the release kinetics is the fastest in the interval 10–20 min. When ammonia-treated surfaces were used, the release of cells was slower than when Ar and O_2_-treated pNIPAAM surfaces were used to initiate detachment, and the difference in release time is even more striking when compared to the control ones. As such, the release is 2–3 times more efficient for the Ar and O_2_ plasma-treated surfaces and 1.5 times faster for those treated with ammonia. This can be explained by the enhanced hydration of the porous surfaces, which could lead to a faster detachment of L929 cells, while the less porous surfaces were slower in the hydration process, leading to a higher detachment time.

The accelerated cell detachment from Ar and O_2_-plasma-treated surfaces could be attributed to the presence of hydrophilic groups on the surface. Therefore, interactions of the polar groups with water are proposed to accelerate surface hydration and decrease the amount of time required for complete cell and cell sheet detachment. For example, in the literature, the presence of charged carboxyl groups based on copolymerizing pNIPAAM with 2-carboxyisopropyl acrylamide, p(NIPAAM-co-CIPAAM) led to controlling/enhancing cell detachment from pNIPAAM-grafted surfaces by manipulating the composition of the grafted polymer, when after 60 min at 20 °C, almost all cells detached from pNIPAAM-coCIPAAM whereas cells only started detaching from pNIPAAM [29]. By plasma functionalization (and/or etching), besides the surface chemistry changes through the introduction of specific chemical groups, the surface morphology, topography, the roughness was also affected, implying a larger presence of pores.

## 4. Conclusions

Plasma treatment in argon, ammonia and oxygen was utilized for the functionalization of pNIPAAM thin films obtained by spin coating in order to tune the cell attachment-detachment processes via the superficial chemical modifications. The results evidenced that argon plasma conducts to a combination of etching/redeposition of pNIPAAM, leading to a less porous, more carboxylic surface. The usage of reactive plasmas generated in oxygen and respectively in nitrogen environment led to surfaces that are more porous and induce an increase of the specific bonds related to the formation of O-C=O and N-C=O bonds, respectively, as demonstrated by XPS investigations. These bonds are also evidenced by the FTIR technique, which indicates the formation of carboxylic acid bonds in the case of oxygen plasma, also noticed for argon plasma, and the amine and amide bonds in the case of ammonia plasma.

These functionalized pNIPAAM surfaces are beneficial for the cells’ adhesion and proliferation, as evidenced by the MTT assays that show at T = 37 °C (above the LCST), viability increased by more than 25% as compared with the pNIPAAM control surfaces (not treated). Moreover, they show a significant increase with respect to the number of cells attached on the substrate, up to 2.8 times and two times more cells being accounted for on the oxygen and argon plasma modified substrate, respectively, as compared to the non-treated one, and a 2–3 times faster detachment when compared to the classical spin-coated pNIPAAM layers. 

This behavior is determined by the hydrophilic groups in the form of carboxylic acid during the oxygen/argon plasma treatment and of amide groups respectively during ammonia plasma treatment, combined with the increased porosity of the layer induced by etching, allowing faster layer hydration below LCST followed by significant swelling impinging the cells from the treated pNIPAAM surface upon surface cooling. 

## Figures and Tables

**Table 1:** Chemical bonds assignment corresponding to pNIPAAM.

	Chemical Bonds	Wavenumber (cm^−1^)
a	C-N bending	1367
b	C-CH_3_ methyl bending II	1386
c	C-CH_3_ methyl bending I	1461
d	N-H bending	1540
e	N-C=O	1646
f	O-C=O	1698
g	CH_3_ symmetric stretching CH_2_ symmetric stretching CH_3_ asymmetric stretching	2874–2970
h	=C-H stretching	3070
i	N-H stretching mode	3308
j	N-H stretching mode	3435

Blue type indicates the bonds corresponding to hydrophobic behavior, while the red type are those related to hydrophilic one.

## Data Availability

The data presented in this study are available on request from the corresponding author.
